# Supplementary material for: Discovery of colossal Seebeck effect in metallic Cu2Se
Source: Nat Commun. 2019 Jan 8;10:72. doi: 10.1038/s41467-018-07877-5 (PMC6325113; doi:10.1038/s41467-018-07877-5)
Supplement: Supplementary file 1 — Supplementary Information [file 41467_2018_7877_MOESM1_ESM.pdf]

Supplementary Information for

Discovery of Colossal Seebeck Effect in Metallic Cu_2Se

Dogyun Byeon[1], Robert Sobota[1], Kévin Delime-Codrin[1], Seongho Choi[1], Keisuke Hirata[1], Masahiro Adachi[2], Makoto Kiyama[2], Takashi Matsuura[2], Yoshiyuki Yamamoto[2], Masaharu Matsunami[1], and Tsunehiro Takeuchi[1*]

Correspondence to: t_takeuchi@toyota-ti.ac.jp

Contents

Supplementary Figure 1 | Seebeck coefficient experimental setup 3

Supplementary Figure 2 | Analysis of raw data –quasilinear regime 4

Supplementary Figure 3 | Analysis of raw data -nonlinear regime.................................. 4

Supplementary Figure 4 | Electrical resistivity temperature dependence in cycles 5

Supplementary Figure 5 | Cycle reproducibility of Seebeck coefficient 5

Supplementary Figure 6 | Detailed electrical resistivity measurements of Cu_2Se 6

Supplementary Figure 7 | Thickness dependence of Seebeck coefficient.......................... 6

Supplementary Figure 8 | Seebeck coefficient and electrical resistivity by TTO 7

Supplementary Figure 9 | Seebeck coefficient measurements of Constantan.................. 8

Supplementary Figure 10 | Seebeck coefficient data of $MnSi_{1.75}$...................................... 8

Supplementary Figure 11 | Sample preparation and treatment....................................... 9

Supplementary Figure 12 | EPMA analysis 9

Supplementary Figure 13 | SEM-EDX analysis... 9

Supplementary Figure 14 | Synchrotron radiation Rietveld analysis 10

Supplementary Figure 15 | Crystal structure model of Cu_2Se α-phase 10

Supplementary Table 1 | Composition of samples..11

Supplementary Table 2 | Rietveld analysis 11

Supplementary Table 3 | Structure parameters...12

Supplementary Note 1 | Seebeck coefficient and electrical resistivity measurement setup 13

Supplementary Note 2 | Analysis of raw data for the Seebeck coefficient.......................14

Supplementary Note 3 | Cycle reproducibility of electrical resistivity and Seebeck coefficient data 15

Supplementary Note 4 | Thickness dependence of Seebeck coefficient............................16

Supplementary Note 5 | Reference comparison of Seebeck coefficient and electrical resistivity 17

Supplementary Note 6 | Reference comparison of Seebeck coefficient data.....................17

Supplementary Note 7 | Sample treatment and chemical analysis detailed.....................18

Supplementary Note 8 | Structural analysis detailed...19

Supplementary Note 9 | First-principles calculations..19

Supplementary References 20

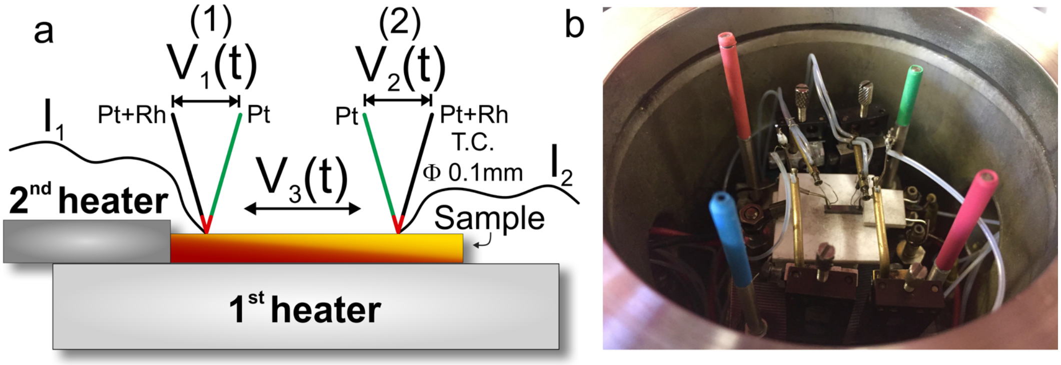

Supplementary Figure 1 | Seebeck coefficient experimental setup

(a) Schematic drawing of Seebeck coefficient experimental setup including primary and secondary heaters and wiring setup. (1) and (2) refer to as terminals for controlling heaters, measuring temperatures, and voltages V_1(t), V_2(t) and V_3(t) as functions of time. (b) The captured image of a typical sample setting in our newly developed measurement system as arranged prior to the measurement. This Seebeck coefficient experimental setup was constructed by ourselves as written in Supplementary Note 1.

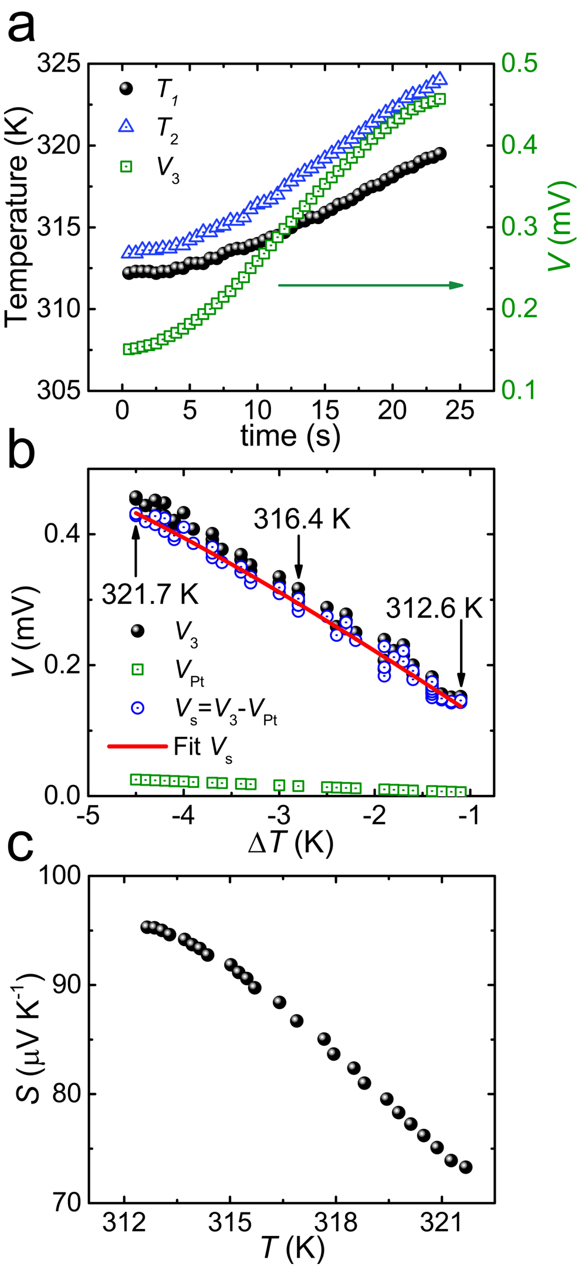

Supplementary Figure 2 | Analysis of raw data –quasilinear regime

(a) Time evolutions of $T_1(t)$, $T_2(t)$ and $V_3(t)$ after applying a temperature gradient by the secondary heater. **(b)** V_3, V_{pt} and V_{samp} as functions of $\Delta T(t)$. **(c)** Seebeck coefficient data deduced from the data in **(b)** plotted as a function of $\bar{T}$.

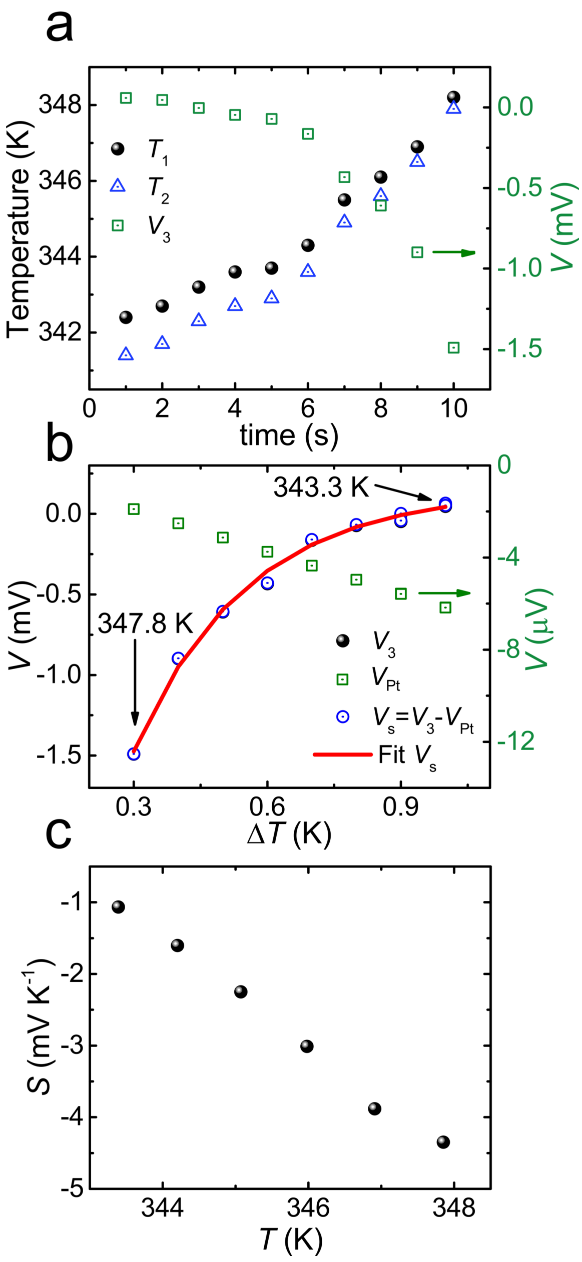

Supplementary Figure 3 | Analysis of raw data -nonlinear regime

(a) Time evolution of $T_1(t)$, $T_2(t)$ and $V_3(t)$ after applying a temperature gradient by the secondary heater. **(b)** V_3, V_{pt} and V_{samp} plotted as functions of ΔT. **(c)** Seebeck coefficient data as a function of $\bar{T}$. The large variation of Seebeck coefficient with a temperature leads to non-linear dependence of $V_{samp}(\Delta T)$ in **(b)**.

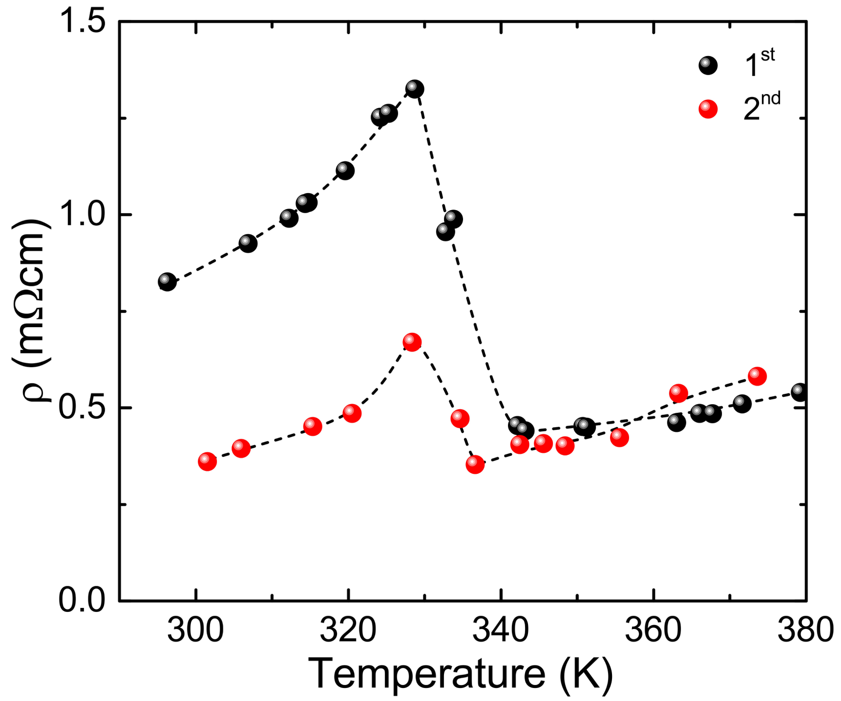

Supplementary Figure 4 | Electrical resistivity temperature dependence in cycles
Electrical resistivity of the single Cu_2Se sample with a thickness of 1.3 mm measured on two cycles.

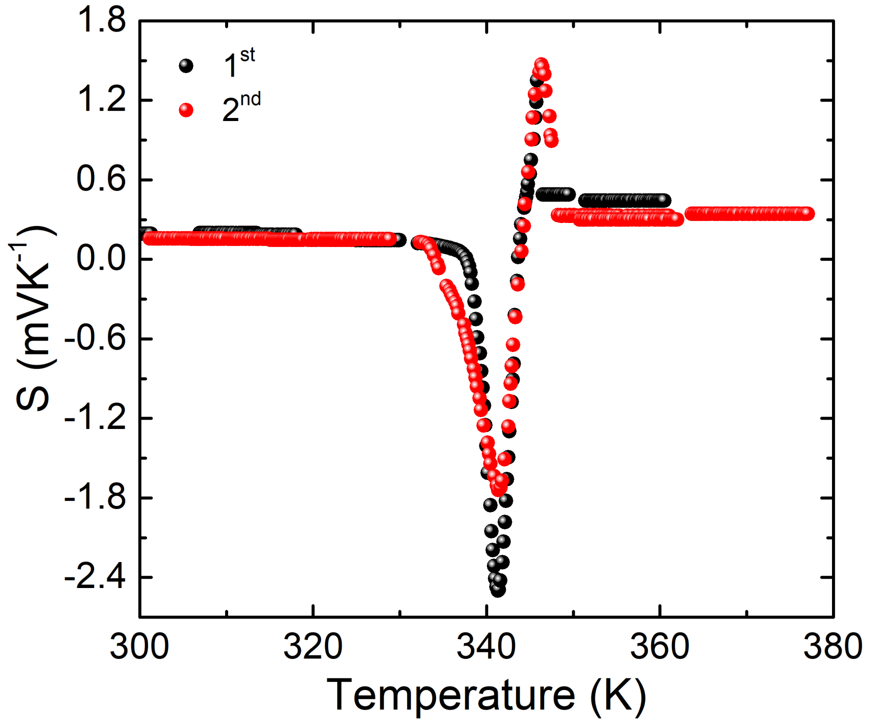

Supplementary Figure 5 | Cycle reproducibility of Seebeck coefficient
Seebeck coefficient of the single Cu_2Se sample with a thickness of 1.3 mm measured on two cycles.

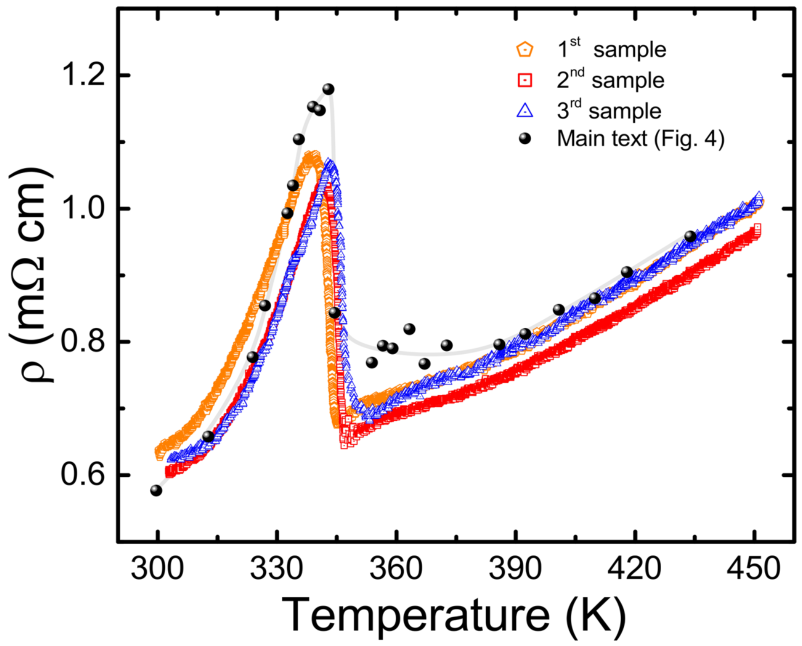

Supplementary Figure 6 | Detailed electrical resistivity measurements of Cu_2Se

The figure displays detailed electrical resistivity measurements of Cu_2Se samples cut from the same ingot (with a thickness of 1.3 mm) on heating together with data comparison shown in the main text as Figure 4a measured by the Seebeck setup.

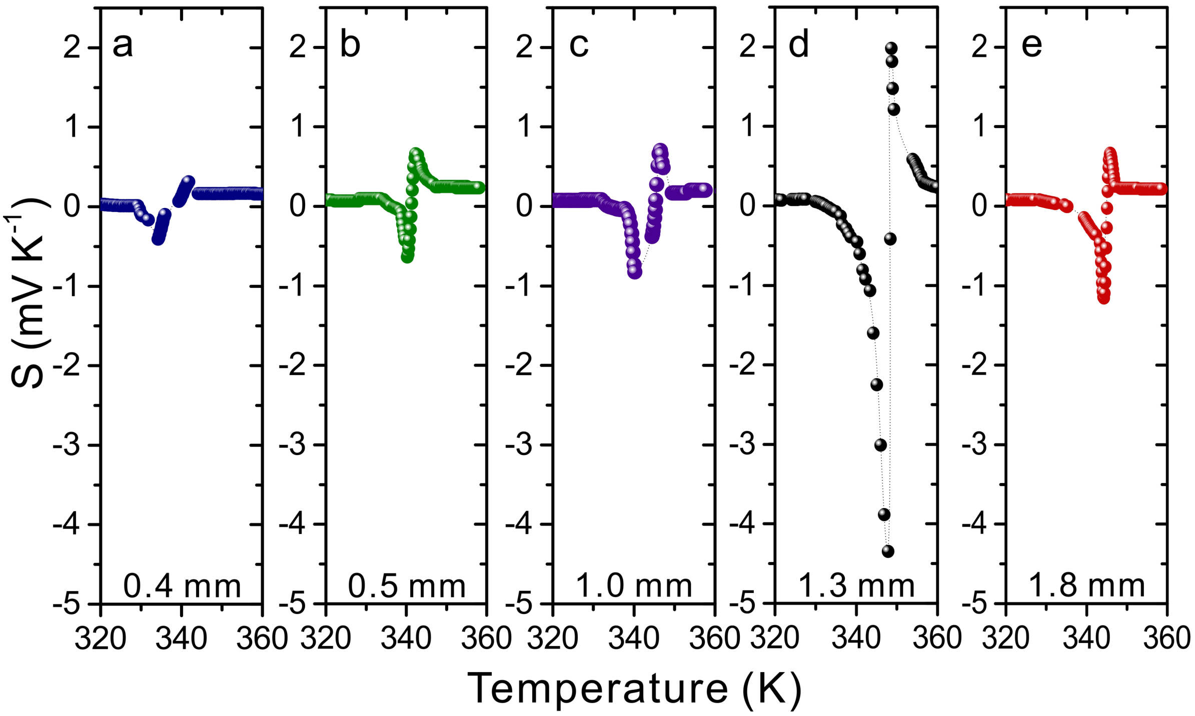

Supplementary Figure 7 | Thickness dependence of Seebeck coefficient

a) - e) represents the Seebeck coefficient data obtained on a single batch of Cu_2Se sample type with various thicknesses in the region of abnormal behavior.

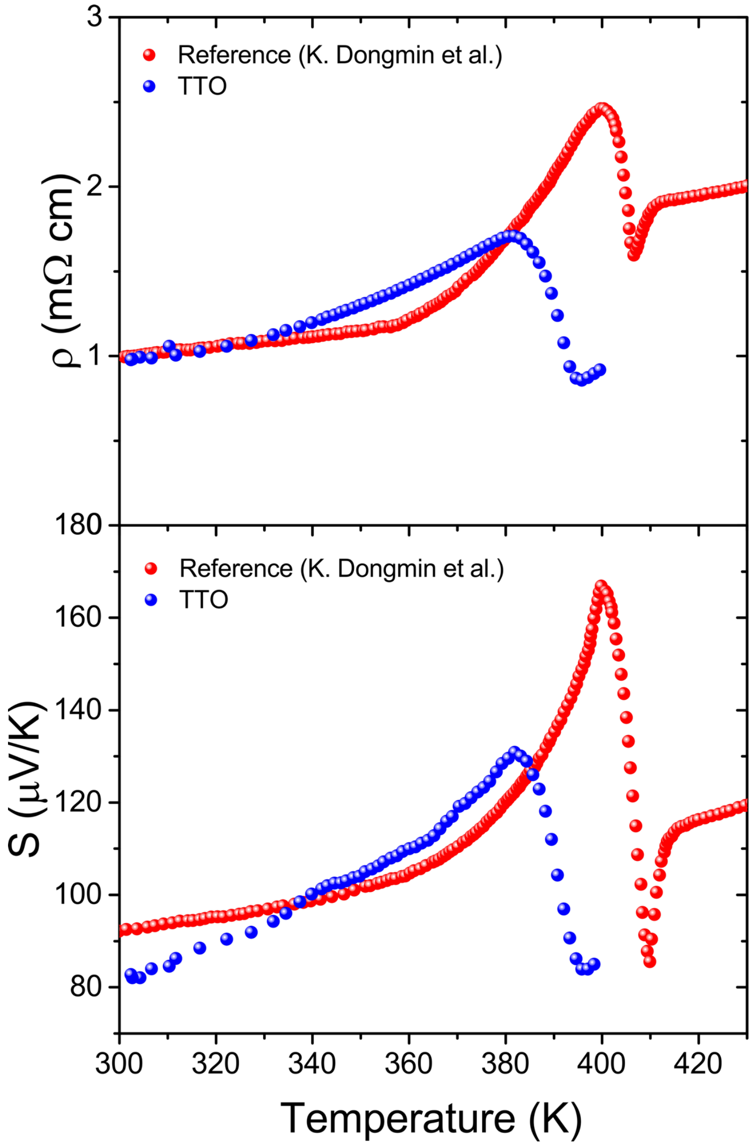

Supplementary Figure 8 | Seebeck coefficient and electrical resistivity by TTO
Electrical resistivity and Seebeck coefficient measured by Thermal Transport Option (TTO) together with reference data.

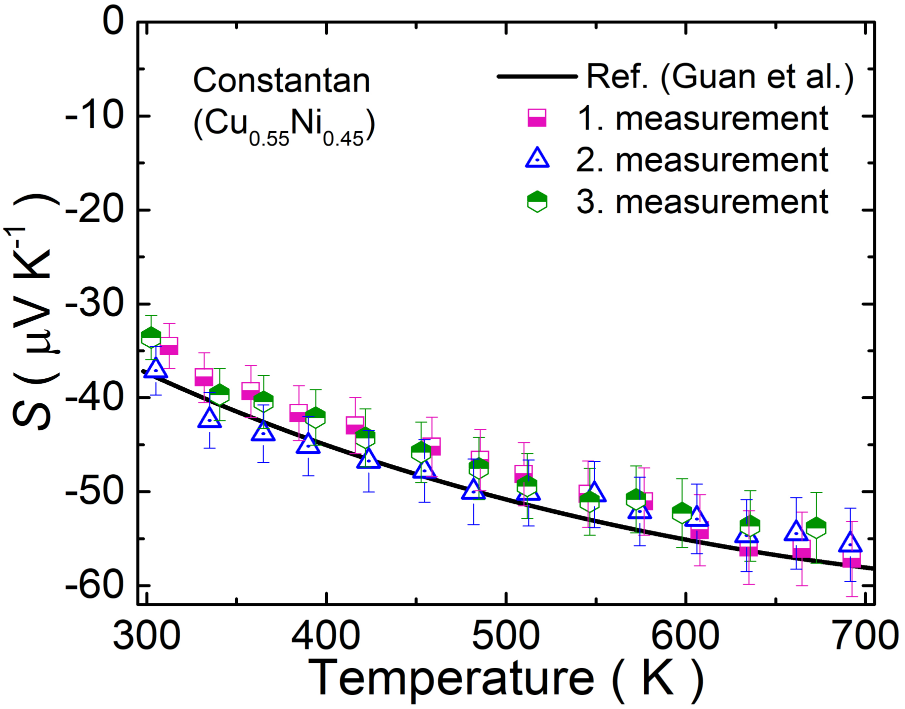

Supplementary Figure 9 | Seebeck coefficient measurements of Constantan

Seebeck coefficient measurements of Constantan ($Cu_{0.55}Ni_{0.45}$) standard measured three times to ensure the validity of the measurement and basic statistics. Each error was calculated as 7 % deviation from its respective value.

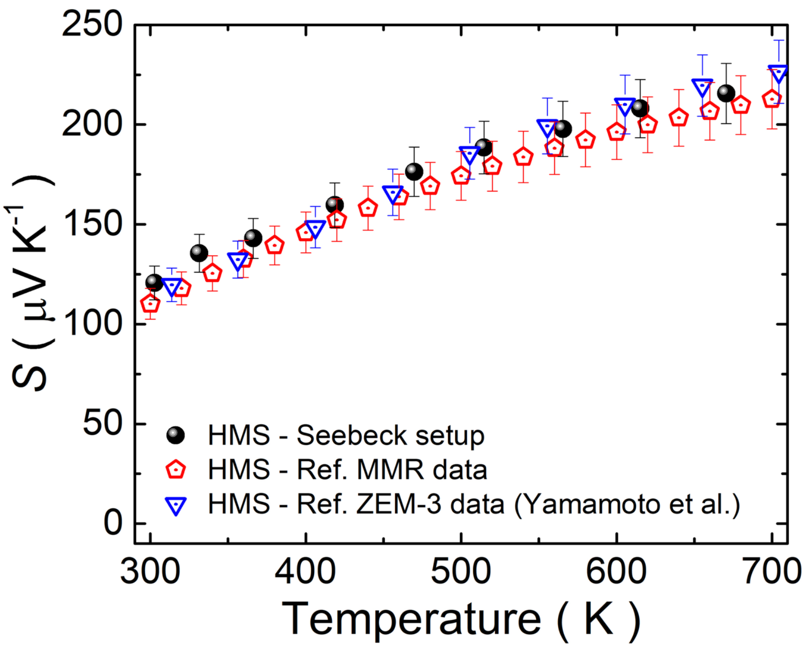

Supplementary Figure 10 | Seebeck coefficient data of $MnSi_{1.75}$

Seebeck coefficient data of $MnSi_{1.75}$ measured by the Seebeck system from MMR technologies, ZEM-3 and our Seebeck setup. Each error was calculated as 7 % deviation from its respective value.

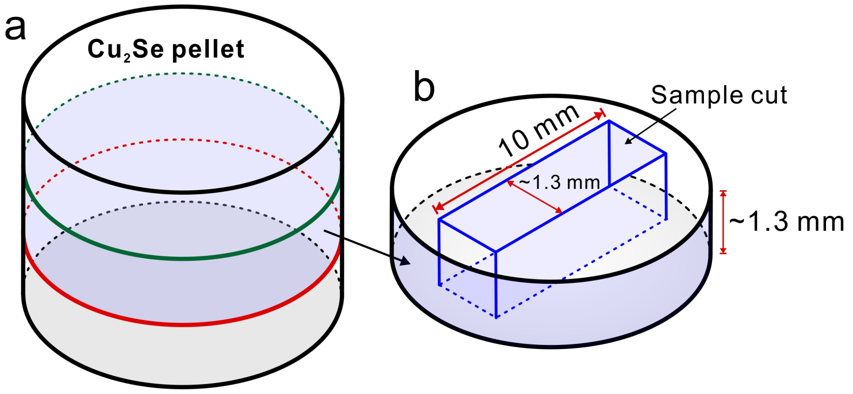

Supplementary Figure 11 | Sample preparation and treatment

(a) As-sintered pellet of Cu_2Se. **(b)** Center sample cut with its dimensions.

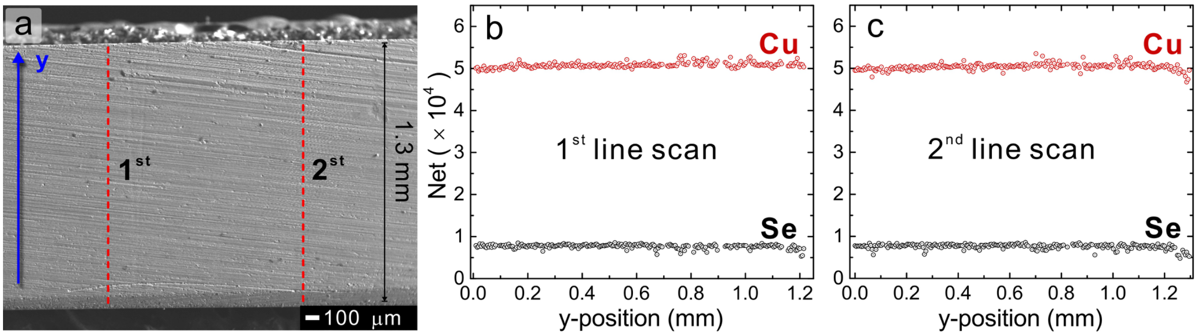

Supplementary Figure 12 | EPMA analysis

(a) Cross-sectional cut of Cu_2Se displaying positions of 2 line EPMA line scans. **(b), (c)** EPMA line scans showing constant relative concentrations of Cu and Se within the vertical y-position.

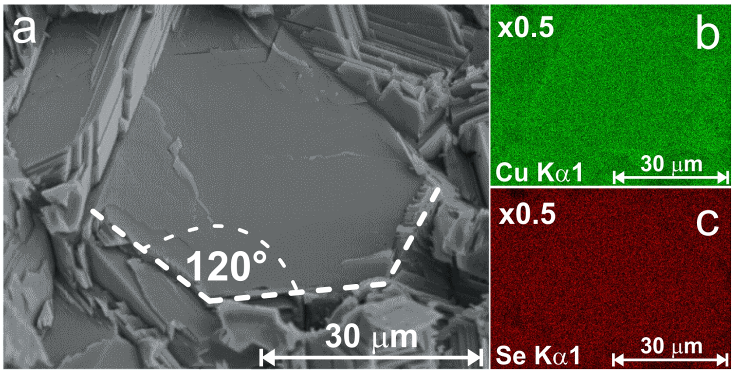

Supplementary Figure 13 | SEM-EDX analysis

(a) SEM image pointing to Hexagonal lattice at room temperature. **(b), (c)** EDX mapping images representing Cu Kα1 and Se Kα1 unveiling the strong homogeneity of Cu and Se

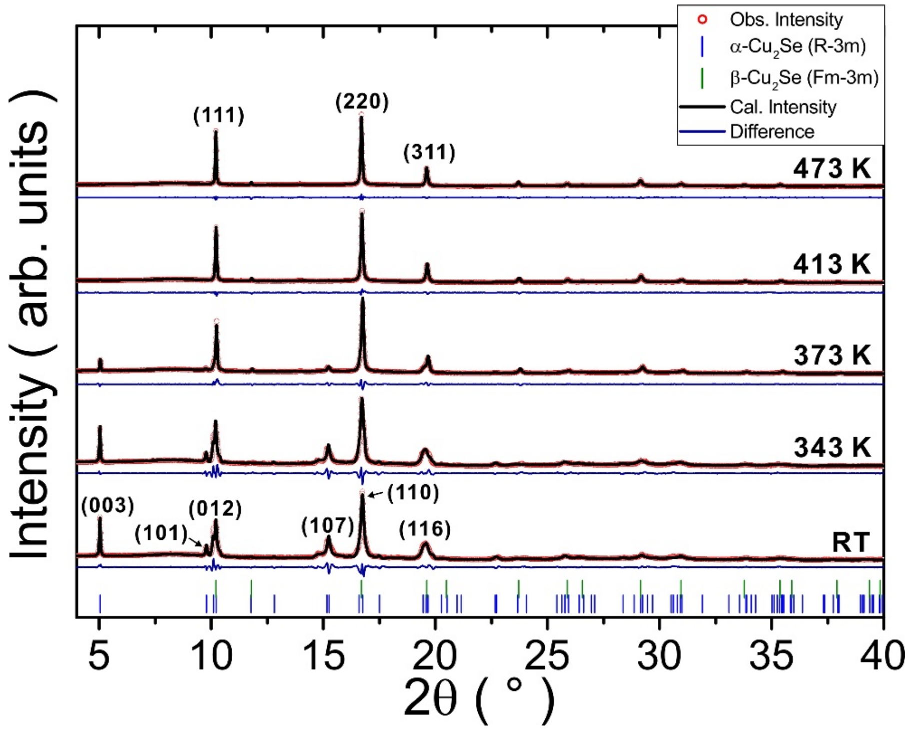

Supplementary Figure 14 | Synchrotron radiation Rietveld analysis
Synchrotron XRD data of Cu_2Se together with Rietveld analysis performed at 30, 70, 100, 140 and 200 °C (343, 373, 413 and 473 K).

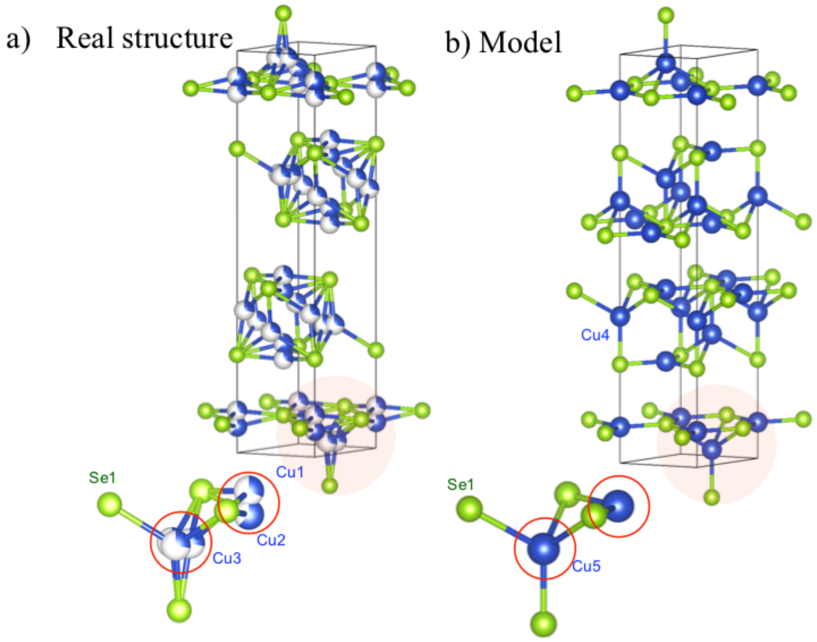

Supplementary Figure 15 | Crystal structure model of Cu_2Se α-phase
(a) The crystal structure of Cu_2Se α-phase. **(b)** The crystal structure model used for the electronic structure calculations. The cluster structures in the shaded areas are magnified in the bottom, where the splitting sites and the combined sites are specified with the open red circles.

Supplementary Table 1 | Composition of samples

	Target material	Nominal composition	Average	Chemical formula
**Cu**	Cu_2Se standard	Cu : Se = 2 : 1	66.67 ± 0.02 at.%	$Cu_{2.000}Se$
**Se**			33.33 ± 0.04 at.%	
**Cu**	Cu_2Se	66.3 ± 0.3 at.%	66.86 ± 0.01 at.%	$Cu_{2.018}Se$
**Se**		33.7 ± 0.2 at.%	33.14 ± 0.02 at.%	

Supplementary Table 2 | Rietveld analysis

Parameters used during Rietveld refinements at 30, 70, 100, 140 and 200 °C including lattice constants, structure factors, phase fractions, and R_p / R_{wp}- fitting factors for both R-3m and Fm-3m.

Room temperature, R_p: 2.14, R_{wp}:3.25, R-3m: 97.93 %, Fm-3m: 2.07 %											
R-3m	x / a	y / a	z / c	B_{iso}	Occ.						
Se1	0	0	0.2437(7)	0.50(8)	1.0						
Cu1	0	0	0.1043(9)	0.32(2)	0.31(6)						
Cu2	0	0	0.0651(7)	0.12(7)	0.61(7)						
Cu3	0.2829(4)	0.5659(1)	0.0205(9)	0.50(1)	0.83(9)						
Lattice parameters		a = 4.1197(6) Å, c = 20.4617(1) Å									
70 °C (343 K), R_p: 2.08, R_{wp}: 3.29, R-3m: 94.32 %, Fm-3m: 5.68 %											
R-3m	x / a	y / a	z / c	B_{iso}	Occ.	Fm-3m	x / a	y / a	z / a	B_{iso}	Occ.
Se1	0	0	0.2448(2)	0.54(7)	1.0	Se1	0	0	0	0.04(2)	1.0
Cu1	0	0	0.1105(4)	0.94(2)	0.31(6)	Cu1	0.25	0.25	0.25	0.07(1)	0.97(2)
Cu2	0	0	0.0666(8)	0.32(5)	0.61(7)	Cu2	0.3390(1)	0.3390(1)	0.3390(1)	0.07(1)	0.79(1)
Cu3	0.2895(4)	0.5784(9)	0.0201(9)	0.76(9)	0.83(9)						
Lattice parameters		a = 4.1232(1) Å, c = 20.4660(3) Å				Lattice parameters		a = 5.7937(4) Å			
100 °C (373 K), R_p: 1.56, R_{wp}: 2.08, R-3m: 29.17 %, Fm-3m: 70.83 %											
R-3m	x / a	y / a	z / c	B_{iso}	Occ.	Fm-3m	x / a	y / a	z / a	B_{iso}	Occ.
Se1	0	0	0.2478(3)	2.37(9)	1.0	Se1	0	0	0	1.12(5)	1.0
Cu1	0	0	0.1136(2)	0.34(5)	0.31(6)	Cu1	0.25	0.25	0.25	1.55(7)	0.97(2)
Cu2	0	0	0.0695(8)	0.52(9)	0.61(7)	Cu2	0.3151(6)	0.3151(6)	0.3151(6)	0.50(1)	0.79(1)
Cu3	0.3049(7)	0.6099(7)	0.0200(8)	3.36(1)	0.83(9)						
Lattice parameters		a = 4.1339(7) Å, c = 20.4200(9) Å				Lattice parameters		a = 5.820(1) Å			
140 °C (413 K), R_p: 1.15, R_{wp}: 1.39, R-3m: 1.73 %, Fm-3m: 98.27 %											
R-3m	x / a	y / a	z / c	B_{iso}	Occ.	Fm-3m	x / a	y / a	z / a	B_{iso}	Occ.
Se1	0	0	0.2478(3)	0.02(5)	1.0	Se1	0	0	0	1.22(7)	1.0
Cu1	0	0	0.1136(2)	0.04(5)	0.31(6)	Cu1	0.25	0.25	0.25	1.63(1)	0.97(2)
Cu2	0	0	0.0695(8)	0.05(4)	0.61(7)	Cu2	0.3163(7)	0.3163(7)	0.3163(7)	0.71(3)	0.79(1)
Cu3	0.2630(1)	0.5260(1)	0.0200(8)	0.05(1)	0.83(9)						
Lattice parameters		a = 4.1305(5) Å, c = 20.4386(9) Å				Lattice parameters		a = 5.8329(1) Å			
200 °C (473 K), R_p: 1.18, R_{wp}: 1.56, R-3m: 0.41 %, Fm-3m: 99.59 %											
						Fm-3m	x / a	y / a	z / a	B_{iso}	Occ.
						Se1	0	0	0	1.34(9)	1.0
						Cu1	0.25	0.25	0.25	1.71(1)	0.97(2)
						Cu2	0.3174(2)	0.3174(2)	0.3174(2)	0.91(1)	0.79(1)
						Lattice parameters		a = 5.8396(4) Å			

Supplementary Table 3 | Structure parameters

Structure parameters of the Cu_2Se α-phase and the crystal structure model used for the electronic structure calculation.

Structure parameters of the Cu_2Se α-phase					
Lattice parameters		*a* = 4.1197 Å, *c* = 20.4617 Å			
Site	Element	*x* / *aa*	*y* / *b*	*z* / *c*	Occ.
Se1	Se	0	0	0.2437	1
Cu1	Cu	0	0	0.1043	0.31
Cu2	Cu	0	0	0.0651	0.61
Cu3	Cu	0.2829	0.6569	0.0205	0.27

Structure parameters of the model used for the electronic structure calculation					
Lattice parameters		*a* = 4.1227 Å, *c* = 20.4490 Å			
Site	Element	*x* / *a*	*y* / *b*	*z* / c	Occ.
Se1	Se	0	0	0.2363	1
Cu4	Cu	0	0	0.0677	1
Cu5	Cu	1/3	2/3	0.0227	1

Supplementary Note 1 | Seebeck coefficient and electrical resistivity measurement setup

A new measurement system of Seebeck coefficient and electrical resistivity has been developed by our group for high-temperature measurements. This apparatus possesses an ability of high-temperature transport property measurements from room temperature up to 750 K. Both resistivity and Seebeck coefficient are measured simultaneously in the same setup. The system consists of a vacuum chamber in which the measurement platform is situated and terminals are further connected to outside measuring unit. The chamber is vacuumed to 10^{-1} Pa maintaining continuous pumping during the whole measurement. The chamber is not subsequently filled with any gas, and thus, only temperature loss being considered in this system can be irradiation and heat flow through the sample platform.

The main part of the system is a platform placed and sealed inside of the vacuum chamber involving two heaters, a sample in between them, and wiring used for measuring temperatures and voltages defining the Seebeck coefficient and resistivity at a particular temperature. The sample is placed next to the secondary heater and onto the primary heater as seen in Supplementary Figure 1a. The real case scenario is represented by a captured image of the as-contacted sample (Supplementary Figure 1b).

In this system, two terminals, (1) and (2), involving two Pt and Pt-13 %Rh wires (Φ 0.1 mm), which are used as both R-type thermocouples (T. C., JIS CI602-1974) and leads for electrical current. Voltages V_1, V_2, and V_3, are measured as continuous functions of time with an increasing temperature, i.e. data sets of $V_1(t)$, $V_2(t)$ and $V_3(t)$ are attained from room to high temperatures continuously, while keeping a temperature gradient between (1) and (2). From the sets, resistivity and Seebeck coefficient can be defined with a very high precision as the data sets are obtained continuously in time. The lead-wires of electrical current have a freedom of their position but, in this study, were placed in the same point of the voltage electrodes because we consider the thin, highly-resistive low-temperature phase on the top of thick, metallic high-temperature phase at the phase transition. With this configuration, the value of power factor is more accurately evaluated for real applications.

We should note here that the Seebeck coefficient is determined by the temperature dependence in a chemical potential of the conduction electrons experiencing the variety of scatterings. Even though we have some unusual temperature gradient in the sample such as that in the present setup, the Seebeck coefficient can be determined precisely, provided that the temperature and the voltage at the contact points are precisely measured.

During a measurement, two temperatures at the defined time are obtained by the thermocouples, and hence, $V_1(t)$ and $V_2(t)$, defining $T_1(t)$ and $T_2(t)$. An average temperature, $\bar{T} = (T_1 + T_2)/2$, and temperature difference, $\Delta T(t) = T_2(t) - T_1(t)$, are defined as a function of time. Subsequently, from $V_3(t)$, Seebeck coefficient at $\bar{T}$ as a function of average temperature $S(\bar{T})$ can be obtained using the following equations.

The measured thermoelectric motive force V is defined as follows

$$V_3 = -\int_{T_1}^{T_2} \left(S_{samp}(T) - S_{Pt}(T) \right) dT. \qquad 1$$

We defined the thermoelectric motive force V_{samp} generated by the sample as

$$V_{samp} = V_3 - \int_{T_1}^{T_2} (S_{Pt}(T))dT \qquad 2$$

After increasing the temperature of the secondary heater, V_{samp} varies as a function of time.

$$V_{samp}(t) = -\int_{T_1+\Delta T_1(t)}^{T_2+\Delta T_2(t)} S_{samp}(T)dT \qquad 3$$

$$V_{samp}(t+dt) - V_{samp}(t) = \Delta V_{samp}(t,dt) = V_{samp}(t+dt) - V_{samp}(t)$$
$$= -\int_{T_1+\Delta T_1(t,dt)}^{T_2+\Delta T_2(t,dt)} S_{samp}(T)dT + \int_{T_1}^{T_2} S_{samp}(T)dT$$
$$= -\int_{T_2}^{T_2+\Delta T_2(t,dt)} S_{samp}(T)dT + \int_{T_1}^{T_1+\Delta T_1(t)} S_{samp}(T)dT \qquad 4$$

We consider both ΔT_1 and ΔT_2 are small enough to make S constant, which is indeed reasonable as we measured $V(t)$ at 0.5 sec intervals. Then the Supplementary equation 4 becomes as

$$\Delta V_{samp}(t,dt) = -S_{samp}(T_2)\Delta T_2(t,dt) + S_{samp}(T_1)\Delta T_1(t,dt). \qquad 5$$

We also assume here that $T_2 - T_1$ is small enough to assume $S_{samp}(T_2) \approx S_{samp}(T_1)$. This assumption is generally valid for a few Kelvin difference between T_1 and T_2. In the circumstance, where S significantly varies with temperature, we have to reduce T_2–T_1 to fulfill the assumption of S equality. In such a case, the condition of $T_2 - T_1 \leq 1.0$ K was used in our measurements. Then the equation develops as

$$\Delta V_{samp}(t,dt) = -S_{samp}(\bar{T})\big(\Delta T_2(t,dt) - \Delta T_1(t,dt)\big). \qquad 6$$

Therefore, the Seebeck coefficient of the sample is defined as follows

$$S_{samp}(\bar{T}) = -\frac{\Delta V_{samp}(t,dt)}{\Delta T_2(t,dt) - \Delta T_1(t,dt)} = -\frac{dV(t,dt)}{d\Delta T_{21}(t,dt)}. \qquad 7$$

In addition to this, pulse alternating current, $I(t)$ with a square pulse amplitude of 10 mA and frequency 0.05 Hz (+10 mA during 10 seconds and -10 mA, 10 seconds) to eliminate any contact resistance and Seebeck contact contributions is applied through (1) and (2) terminals. By using this approach, resistivity as a function of time, R(t), and thus, the resistivity at an average temperature $R(\bar{T}) = (R_+(\bar{T}) + R_-(\bar{T}))/2$ is attained, where $R_\pm = V_\pm(\bar{T})/I_\pm$.

Supplementary Note 2 | Analysis of raw data for the Seebeck coefficient

The standalone measurement, as well as an analysis of raw data obtained from our Seebeck setup, is carried out in several steps. As mentioned in Supplementary Note 1, raw data include voltage sets attained in time at a particular temperature $V_1(t)$, $V_2(t)$ and $V_3(t)$ which provide us with $T_1(t)$ and $T_2(t)$, through R-type thermocouples, of (1) and (2) terminals and

V_3(t) measured between those terminals used for determination of the Seebeck coefficient and resistivity. The whole measurement is performed as follows.

At first, the primary heater is used to heat the sample to a target temperature of interest. Subsequently, the secondary heater is turned on so that the temperature gradient is applied. The $T_1(t)$, $T_2(t)$ and $V_3(t)$, as well as temperature gradient, are gradually increased with increasing time (Supplementary Figure 2a). $T_1(t)$ and $T_2(t)$ are used to determine $\bar{T}(t)$ and $\Delta T(t)$. The thermoelectric motive force of Pt, V_{pt}(t), was removed from $V_3(t)$ to obtain solely sample voltage contribution to the Seebeck coefficient data $V_{samp}(t) = V_3(t) - V_{pt}(t)$ as Supplementary equation 2. Then, we plot V_{samp} as a function of corresponding ΔT as shown in Supplementary Figure 2b. The Seebeck coefficient is determined as a derivative of V_{samp} with the respect to ΔT and plot at the corresponding $\bar{T}$ as in Supplementary Figure 2c. By this method, we obtain the Seebeck coefficient in a narrow temperature interval of a few of Kelvin since at every target temperature, by measuring $T_1(t)$ and $T_2(t)$ many temperatures $\bar{T}(t)$ and Seebeck data points are acquired.

Worth noting that in the case of Supplementary Figure 2a-c, a quasilinear regime of $V_{samp}(t)$ is shown. It is called quasilinear on purpose because the Seebeck coefficient is almost constant and vary only about 22 μV/K within the temperature range of 9 K, which indeed is a very small variation when compared to the other regime where the unusual behavior is observed. On the other hand, for any other purposes, we can definitely say that this is a linear behavior with a very small alternation in $S(T)$.

For completeness, in Supplementary Figure 3a-c, we show the same analysis of V_{samp}(t) in a nonlinear regime. In a nonlinear regime as well as the linear case, fitting is used to improve the data quality and decrease a noise level in derivative curves, and hence, Seebeck coefficient data. We surely confirmed that this procedure does not affect the overall outcome of the Seebeck coefficient.

Supplementary Note 3 | Cycle reproducibility of electrical resistivity and Seebeck coefficient data

Cycle reproducibility of the presented results shown in the main text was carefully investigated. Both electrical resistivity and Seebeck coefficient of one particular sample with a thickness of 1.3 mm were simultaneously measured by our setup in cycles (Supplementary Figure 4). The time between each cycle was set to be more than 30 min to ensure complete relaxation of the setup as well as the mounted sample.

As can be seen, at first, the resistivity of the as-prepared sample shows the same behavior as previously reported and described in the main text, namely, an increasing behavior in the range of the phase transition followed by a sudden drop to about 0.45 mΩcm being a linearly but shallowly increased subsequently. However, after the cooling down and measuring the second time, the absolute value of resistivity at room temperature and in the temperature range of the phase transition decreased more than two times. Interestingly, the resistivity was measured to be almost identical above the phase transition and its temperature did not change after the first measurement. The sudden reduction in resistivity values can be explained by the non-complete transformation of the sample to the low-temperature phase at room temperature

even after the relaxation, and the presence of high-temperature phase after the first heating as the high-temperature phase is much more conductive. This means that each cycle might change the phase fraction at room temperature to be slightly different and has an influence on the overall resistivity in our samples.

Simultaneously measured Seebeck coefficient (Supplementary Figure 5) shows similar development. Although the abnormal behavior effect, i.e., the magnitude of the negative peak of the Seebeck coefficient decreases with the cycles, the phenomenon sustained at the phase transition as presented in the main text. The fact that the decrease of the magnitude can be actually seen, is caused by the phase fraction alternation within the sample after the first measurement as it was observed in the case of electrical resistivity. This means that the number of measurement cycles has the nonzero influence on the phase fraction inside of the sample strongly supporting our scenario that the coexistence of both phases leads to the abnormal behavior of the Seebeck coefficient.

To more profoundly confirm the extremely high measured large values of power factor as presented in this work, the more detailed measurements of electrical resistivity were performed on Cu_2Se sample type of the same batch (with a thickness of 1.3 mm) on heating in full temperature range by the Seebeck setup. Since our experimental setup of simultaneous measurement of Seebeck coefficient and electrical resistivity prevented us from measuring electrical resistivity with tiny temperature steps, these measurements were performed without the Seebeck measurement. As shown in the Supplementary Figure 6, the additional measurements on three different samples cut from the same ingot display the consistent results with that displayed in Fig. 4a of the main text without neither any unusual artifact nor abnormal behavior. Thus, the extraordinary power factor acquired is certainly verified. We can see that even though a small dispersion of resistivity values is obtained, these values are almost negligible to be important. More interestingly, by using the displayed values of electrical resistivity, and assuming the same Seebeck coefficient along with the thermal conductivity, the power factor would be increased in this temperature region.

Supplementary Note 4 | Thickness dependence of Seebeck coefficient

If the abnormal behavior of Seebeck coefficient discovered in the Cu_2Se system is caused by a coexistence of both phases in the narrow temperature range and the vertical temperature gradient, the thickness of particular sample should have a nonzero effect on the Seebeck data and optimum of the phenomenon should be observable. This was studied via thickness dependence of the Seebeck coefficient measured on a single batch of Cu_2Se samples prepared by the same method and chemical composition carefully polished to the desired thicknesses (Supplementary Figure 7a-e).

It can be evidently visible that the thickness, d, has a strong impact on the overall behavior of the Seebeck coefficient data. The abnormality in the Seebeck data is almost minimal for d = 0.4 mm and with increasing d, Seebeck coefficient sign change is more pronounced reaching the thickness with the effect of most dominance at $d \approx 1.3$ mm followed by the decreasing presence of the behavior with further increasing of d. This dependence obviously adopts our scenario as by using the same experimental setup and measuring protocol,

a two-phase region of coexistence ought to change significantly and the visibility of abnormal behavior should vary, which is indeed clearly visible, and confirms the arguments presented in this work.

Supplementary Note 5 | Reference comparison of Seebeck coefficient and electrical resistivity

The abnormal behavior of the Seebeck coefficient of our prepared Cu_2Se samples was additionally investigated by a commercially available Physical Property Measurement System (PPMS) designed by Quantum Design, Inc. The option used for the measurement was Thermal transport option (TTO) by which both resistivity and Seebeck coefficient were measured simultaneously in a temperature range of 300 – 400 K in the high vacuum mode. The sequence of measurement with a temperature increase was set to be 0.2 K/min in 4 steps, 0.1 K/min in 2 steps and 0.05 K/min in 30 steps for 300 – 330 K, 335 – 340 K, 342 – 400 K ranges, respectively. The temperature gradient applied through the sample used for Seebeck coefficient measurement was set automatically by PPMS to be about 1 K. In Supplementary Figure 8, both resistivity and Seebeck coefficient data measured by the apparatus are shown. The data measured on Cu_2Se sample by commercially available system effectively match to the reference data[1], though, some discrepancy between our measured and reference data can be observed. This can be explained by stoichiometry aberration as it was proved that a very small difference in the composition has a significant influence on the transport properties. More interestingly, by using the commercially available system no abnormal behavior nor any sign change in the Seebeck data was observed. This is surely caused by the different measurement system used having different heating geometry, and thus, influencing the heat distribution. As already mentioned, in our case, two heaters leading to a combination of horizontal and vertical temperature gradients instead of unidirectional heating are used. This proves that the heat distribution used in our Seebeck system and employment of presented analysis has significant effect onto the unveiling of the abnormal Seebeck coefficient behavior, which cannot be detected by the other method as investigated before in Cu_2Se system.

Supplementary Note 6 | Reference comparison of Seebeck coefficient data

To confirm this unusual behavior of Seebeck coefficient as presented in the main text measured by our newly developed Seebeck coefficient apparatus, we had to exclude any possible mistake related to the experimental setup, which might provide us with wrong data. In order to do so, we confirmed the validity of our experimental setup by measuring a reference sample of Constantan ($Cu_{0.55}Ni_{0.45}$) standard. In Supplementary Figure 9, several and typical measurement of constantan Seebeck coefficient from 300 to 700 K using the Seebeck setup to avoid any likelihood of statistical error together with reference data[2] are shown. As clearly seen, within 7 % error, the Seebeck coefficient data are same in the whole temperature interval as those of reference data without any anomaly, and hence, the data obtained from our setup should be real.

To ensure the validity of our experimental data obtained from the Seebeck setup, another already widely established material with commonly known values of Seebeck coefficient is Higher Manganese Silicide (HMS), $MnSi_{1.75}$ in particular, used for the comparison[3]. In Supplementary Figure 10, we plot $MnSi_{1.75}$ measured by three different systems in a temperature range from 300 to 700 K. The presented data have been carefully measured by standard measurement systems such as ULVAC ZEM-3 and the Seebeck measurement system from MMR technologies Co. with a comparison of HMS measured in our Seebeck system. For completeness, HMS Seebeck coefficient data measured by MMR were obtained additionally as the control measurement by our group. As seen in the plot, the data are almost identical within the error bar of less of 7 %, and thus we conclude that the unusual behavior of Seebeck coefficient presented in the main text obtained from our Seebeck setup are truly valid and believable without any doubts.

Supplementary Note 7 | Sample treatment and chemical analysis detailed

In our study, conventional self-propagating high-temperature synthesis (SHS) method was used to prepare Cu_2Se samples[4-7]. By this method, we obtained sintered pellets being subsequently cut in the middle to ensure homogeneity as we found out that the Cu and Se concentrations slightly vary across the whole pellet due to SPS process and ionic conduction in high-temperature phase and the center position shows the same composition. Later on, the cuts were formed to a sample with a length, width and height of 10 mm, 1.3 mm and 1.3 mm, respectively, as shown in Supplementary Figure 11. These cuts were used to investigate structural and chemical as well as their thermoelectric properties.

Before extracting a pellet cut to a sample shape, chemical analysis by means of EPMA was performed at room temperature on both a fractured Cu_2Se sample surface prepared by our laboratory and Cu_2Se standard provided by Sigma Aldrich (Cu_2Se (99.95 %), Sigma Aldrich, CAS 20405-64-5, P.N.: 481629-5G). Supplementary Table 1 shows typical concentrations of Cu and Se obtained on our Cu_2Se samples together with a comparison with the Cu_2Se standard. The chemical composition is obtained from 10 randomly but evenly distributed point scans chosen on the surfaces of both the standard and our pellet, where EPMA spectra were taken by four different detectors, and combined to obtain relative concentration of Cu and Se. The chemical formulae of both Cu_2Se standard and our prepared sample type are shown in the table as well. Typically, the samples are Cu-rich due to higher volatility of Se, though the off-stoichiometry is in error of less than 2 %, and thus we can assume the target material to be reached.

As can be clearly seen, the stoichiometry is fulfilled in both cases as shown by quantitative EPMA point analysis within 2% error, thus we can confirm the stoichiometry and homogeneity from the top of the Cu_2Se pellet. To confirm, vertical chemical uniformity, the pellet was cut along a diameter of the pellet and cross-section line scans using EPMA were performed, as shown in Supplementary Figure 12a-c. Two scans avoiding possible statistical mistake are displayed. Since the relative concentration of Cu and Se within our sample cuts used for thermoelectric analyses is almost constant, we can conclude that vertical chemical homogeneity is also satisfied.

Additional chemical analyses using SEM-EDX were carried out at room temperature on a fractured Cu_2Se sample surface to confirm spatial homogeneity of our samples. As can be seen in Supplementary Figure 13a-c, samples display exceptionally good chemical homogeneity of Cu and Se without neither segregation of elements nor the presence of precipitates measured by SEM-EDX mapping. The ratio of Cu and Se, and thus stoichiometry, was the identical as in the case of EPMA again confirming the homogeneity of the Cu_2Se samples. Moreover, as seen by XRD measurements, SEM image also verifies the hexagonal crystal structure with an archetypal grain size of approximately 20 – 30 μm (Supplementary Figure 13a) meaning that room temperature refinement using hexagonal (*R*-3*m,* Pearson Symbol: hR6) crystal structure is correct.

Supplementary Note 8 | Structural analysis detailed

Structural investigations of the prepared Cu_2Se samples were carried out at Aichi Synchrotron Radiation center (Beamline BL5S2) used for XRD. Before the measurements, fine powder Cu_2Se was prepared in an agate mortar using smooth grinding in ethanol and dried afterward. The powder was carefully inserted into borosilicate glass capillaries with a diameter of 0.2 mm sealed at room temperature. The XRD experiments were performed in Debye-Scherrer geometry using X-ray beam with an energy of ~20.7 keV corresponding to a wavelength, λ, of about 0.6 Å. The exact λ value was determined by Rietveld analysis on the CeO_2 standard used for calibration before Cu_2Se experiments. The measurements were performed at room temperature, and temperature steps of 343, 373, 413 and 473 K, as used in the main text, reflecting 70, 100, 140 and 200 °C, respectively. Each scan lasted 6 min per the whole 2θ interval ensuring sufficient statistics. Rietveld analysis on obtain data was subsequently performed.

It was found that, at room temperature, XRD pattern consists solely of the low-temperature phase: hexagonal crystal (Pearson Symbol: hR6) with space group: *R*-3*m.* The low-temperature phase Cu_2Se undergoes a smooth phase transition to a cubic high-temperature phase (Pearson symbol: *cF*12), that has a crystal structure of *Fm*-3*m* symmetry, over a high-temperature interval from 320 K to 390 K. The contribution of high-temperature phase is gradually increased with an increasing temperature. As seen in Supplementary Figure 14, all patterns were well-refined using both hexagonal (low-temperature) and face-centered cubic (high-temperature) phases at each temperature. All refinement parameters including lattice constants, structure factors, phase fractions, and R_p / R_{wp}- fitting factors for both *R*-3*m* and *Fm*-3*m* can be found in Supplementary Table 2.

Supplementary Note 9 | First-principles calculations

For calculating the electronic structure and Seebeck coefficient of Cu_2Se low-temperature α-phase, we employed a simplified model of the crystal structure. The copper sites in α-phase are split into several positions of the vibration center to prevent us from easily calculating the electronic structure. Therefore, we removed these split sites from the model.

The structures of the real sample and the model are schematically drawn in Supplementary Figure 15a and b, and the structure parameters are summarized in Supplementary Table 3.

The structure optimization was performed with VSAP using the pseudo-potential method. For the electron correlation, we employed the Generalized Gradient Approximation developed by Perdew, Burke, and Ernzerhof (GGA-PBE)[8]. We used 55 $\boldsymbol{k}$-points generated on a mesh of $10 \times 10 \times 2$ in the irreducible wedge of the first Brillouin zone with the energy cutoff of 400 eV.

Another program, WIEN 2K, which uses the full potential linearized augmented plane-wave (FLAPW) method, was used for obtaining the detailed electronic structure. The electron correlation was taken into account by GGA-PBE. In the irreducible wedge of the first Brillouin zone, 110 $\boldsymbol{k}$-points on a mesh of $10 \times 10 \times 10$ were used for the calculation. The obtained electronic structure was used with BoltzTraP in order to calculate the Seebeck coefficient at 345 K as a function of chemical potential.

Supplementary References

1. K. S. Dongmin *et al.*, Apparent critical phenomena in the superionic phase transition of $Cu_{2-x}Se$. *New J. Phys.* **18**, 013024 (2016).
2. A. Guan *et al.*, An experimental apparatus for simultaneously measuring Seebeck coefficient and electrical resistivity from 100 K to 600 K. *Rev. Sci. Instrum.* **84**, 043903 (2013).
3. A. Yamamoto *et al.*, Thermoelectric properties of supersaturated Re solid solution of higher manganese silicides. *Jpn. J. App. Phys*. **55**, 020301 (2016).
4. X. Su *et al.*, Self-propagating high-temperature synthesis for compound thermoelectrics and new criterion for combustion processing. *Nat. Commun.* **5**, 4908 (2014).
5. H. C. Yi, J. J. Moore, Self-propagating high-temperature (combustion) synthesis (SHS) of powder-compacted materials. *J. Mater. Sci.* **25**, 1159-1168 (1990).
6. G. Gershinsky *et al.*, Ultra fast elemental synthesis of high yield copper Chevrel phase with high electrochemical performance. *J. Solid State Chem.* **188**, 50-58 (2012).
7. A. G. Merzhanov, The chemistry of self-propagating high-temperature synthesis. *J. Mater. Chem.* **14**, 1779-1786 (2004).
8. J. P. Perdew, K. Burke, M. Ernzerhof, Generalized Gradient Approximation Made Simple. *Phys. Rev. Lett.* **77**, 3865-3868 (1996).
